# The impact of lean management on frontline healthcare professionals: a scoping review of the literature

**DOI:** 10.1186/s12913-021-06344-0

**Published:** 2021-04-26

**Authors:** Zeyad Mahmoud, Nathalie Angelé-Halgand, Kate Churruca, Louise A. Ellis, Jeffrey Braithwaite

**Affiliations:** 1 Australian Institute of Health Innovation, Macquarie University, Level 6, 75 Talavera Rd, Sydney, NSW 2109 Australia; 2 Université de Nantes, LEMNA, F-44000 Nantes, France; 3 Université de la Nouvelle Calédonie, LARGE, Nouvelle Calédonie, France

**Keywords:** Lean management, Health care, Staff outcomes, Healthcare professionals, Literature review

## Abstract

**Background:**

Lean management practices are increasingly used in hospitals. However, their impacts on staff have not been systematically synthesised. This scoping review aims to synthesise the evidence on the effects of Lean Management practices on frontline healthcare professionals.

**Methods:**

A search was conducted in February 2020 on multiple databases to identify relevant sources. Studies had to satisfy the following inclusion criteria to be considered: published in English or French, peer-reviewed, empirical, studied the use of Lean in a healthcare setting and focused on its impacts on frontline workers. The studies included were heterogeneous in terms of participants. Findings were coded and classified using a thematic analysis. The quality and methodological rigour of the reviewed articles were assessed to establish a level of confidence in their findings.

**Results:**

Of 998 identified articles, 17 were included in the review. The findings were coded into four themes: (1) Morale, motivation and job satisfaction (*n* = 9, 2) work intensification, job strain, anxiety, stress and dehumanisation (*n* = 7, 3) teamwork, communication and coordination (*n* = 6); and (4) learning, innovation and personal development (*n* = 3). Overall, the articles reported positive (*n* = 11), negative (*n* = 3) and mixed (*n* = 3) impacts of Lean on frontline healthcare professionals.

**Conclusion:**

This review is the first to synthesise and highlight the gaps in the existing literature examining the impacts of Lean on frontline health professionals. The review revealed a range of both positive, negative and mixed effects, and points to the need for more empirical research to identify the underlying reasons leading to these outcomes.

**Supplementary Information:**

The online version contains supplementary material available at 10.1186/s12913-021-06344-0.

## Background

The basic premise of Lean Management (LM)—which has its origins in the automotive industry—is that greater efficiency can be achieved through a process of continuous improvement aimed at eliminating waste and maximising value-adding activities [1–4]. Also referred to as the Toyota Production System, or TPS, LM constitutes a radical transformation of traditional mass-production methods [3, 4]. Instead of focusing on producing large volumes of standardised goods, LM emphasises waste elimination as a way of improving the flexibility of productive resources and addressing variability in customer demands [1].

It is challenging to retain a singular and straightforward definition of LM and what it encompasses due to the vast discrepancies between the definitions used by various authors writing on the subject [5]. However, for the purpose of this review, we use Radnor et al’s definition of Lean as a “management practice based on the philosophy of continuously improving processes by either increasing customer value or reducing non-value adding activities (muda), process variation (mura), and poor work conditions (muri)” [6]. Generally, LM is considered to be the “antidote” to waste in organisations [3]. Waste, defined as tasks and processes that do not contribute to the creation of value but consume organisational resources, is associated with inefficiencies, reduced flexibility and the generation of unnecessary costs [3]. Ohno, the developer of LM, identified seven sources of waste (summarized in Table 1) and pioneered managerial and organisational tools and techniques to help organisations get rid of them (e.g., Value Stream Mapping, 5S, Kanban, Standardisation, Process map) [2]. While the tools of Lean are numerous, they are broadly designed to help organisations understand their customer needs, identify the value-adding activities essential to producing services and products desired by their customers, create production flow by reducing unnecessary delays and interruptions, reduce inventory and overproduction, and continuously improve and refine their productive processes [8].

**Table 1:** Ohno’s seven sources of waste in organisations

Type of Waste	Definition	Example
**Overproduction (OPN)**	Parts are manufactured without any new order or demand from customer. OPN leads to excessive work in process stocks.	Large batch size, unstable schedule, unbalanced cells, inaccurate information on demand.
**Excess inventory**	Storage of products with no order on hand.	Excess inventory, large batch size, long change over time.
**Waiting**	Idle time for machines or workers due to bottlenecks of ill-planned production flow.	Long changeover, unreliable process, time required to perform re-work.
**Motion**	Unnecessary motions of workers, which divert them from actual processing work. Motion involves poor ergonomics of production.	Poor layout, poor method design, large batch size, poor workplace organisation.
**Transportation**	Movement of materials that do not add any value to the product.	Poor layout, large batch size, multiple storage locations.
**Over-processing**	Unintentional conduct of more processing work than warranted by customer requirement.	No standardisation of ideal techniques, unclear specification, or quality acceptance standards.
**Defects**	Production with incorrect specifications, physical defects leading to increase in cost.	Inadequate training, skill shortage, operator error, excessive stock.

Source: P Arunagiri and A Gnanavelbabu [7]

It did not take long for LM to migrate from Toyota and the car manufacturing industry into service-delivery organisations and then public institutions [5, 9–11]. LM has indeed been linked to a host of positive organisational outcomes across-the-board, including improved quality of goods and services, reduced costs and increased productivity [12]. In healthcare, evidence of LM implementation can be found on the micro (operational), meso (strategic) and macro (policy) levels. It has been implemented in a variety of settings including operating rooms, emergency departments, mental health centres, pharmacy services, information departments, and ambulatory care clinics [13, 14]. Overall, LM has been associated with reduced waiting times in emergency departments [15, 16], fewer medical errors [17], and improved clinical pathways [18].

In healthcare, previous literature reviews have identified different approaches to LM implementation [13, 19–21]. On one hand, LM is considered as a comprehensive organisational philosophy aimed at systematically addressing waste at all levels [21]. On the other hand, LM is seen as a toolbox with organisations often implementing one or two LM practices to address waste in a single process or on a small scale (e.g., one ward, or a specific unit) [8]. Whilst piecemeal implementation of LM could be effective in reaching desired performance and efficiency goals, there is little evidence on the long-term sustainability of such gains [19]. In particular, these approaches often overlook crucial elements of LM implementation such as employee engagement and participation.

Against a backdrop of increasingly scarce human, material and financial resources [14], LM has rapidly grown in popularity amongst health practitioners and managers interested in improving the efficiency of their services [22]. It is within this context of proliferation, that we set out to investigate how health professionals experience LM practices and are impacted by them. This is particularly important in light of recent conceptual developments calling for a more holistic approach to the adoption of LM which takes into account both technical and people-oriented strategies [20, 21, 23]. As demonstrated by a growing number of reviews, going beyond the technicalities of LM is a key factor in its successful implementation within organisations [19, 24]. Addressed to policymakers, managers, quality improvement personnel and researchers, this review aims to identify articles addressing the effects of LM on the health workforce and characterise the impacts discussed therein. This scoping review was guided by the following research question:

RQ1: What are the impacts of LM interventions on frontline healthcare professionals?

## Methods

A scoping review was conducted to gain a deeper understanding of how LM impacts frontline healthcare professionals. Findings of the review are reported in accordance with the PRISMA-SCR guideline [25]. This choice of methodology is justified by the emerging nature of the evidence on the impact of LM on the health workforce [26]. The aim of this review is to provide a comprehensive overview of the existing literature and put forward a research agenda for future research on this topic. With that aim in mind, a review strategy was developed and approved by all members of the research team prior to systematically searching the following academic databases in February 2020: Scopus, Emerald, EBSCO business premier and PubMed. The search strategy was not published or registered in an open platform. The choice of databases allowed for the identification of relevant publications in the fields of health science, as well as management studies where LM originated. Papers were searched by combining a set of topic-related keywords (Lean approach, Lean process, Lean method, Lean transformation, Lean philosophy, Lean principles, Lean practices, Lean process improvement, Lean management, Lean healthcare, Lean thinking, Lean production, Lean six sigma, Toyota production system) and a group of setting-related keywords (health care, healthcare, hospital). Only peer-reviewed articles were searched; news articles, conference proceedings, magazines, trade publications and book chapters were excluded using the exclusion parameters of the online databases during the search phase. No starting date was specified, and articles published up to 29 February 2020 were included. Table 2 portrays the use of the search strategy using Scopus database as an example. Additional file 1 includes an example of the search string used to query PubMed and Scopus.

**Table 2:** Search strategy used in Scopus database

Constructs	Search terms
**The topic (Lean Management)**	“Lean approach” OR “Lean process” OR “Lean method” OR “Lean transformation” OR “Lean philosophy” OR “Lean principles” OR “Lean practices” OR “Lean process improvement” OR “Lean management” OR “Lean healthcare” OR “Lean health care” OR “Lean thinking” OR “Lean production” OR “Lean Six Sigma” OR “Toyota production system”
**AND**	
**The setting (Healthcare)**	healthcare OR “health care” OR hospital

Search results from each of the databases were aggregated and imported into an Endnote library, and duplicate entries were removed. Abstracts had to satisfy the following inclusion criteria (Table 3) to be considered in the review: published in English or French, peer-reviewed, empirical, studied the use of LM (i.e., reporting on the use of at least one LM activity) in a healthcare setting (i.e., any facility where healthcare services are delivered) and focused on its outcomes on frontline healthcare workers (i.e., with the primary aim of reporting impacts or effects of LM on staff working at a healthcare setting). Articles discussing the experiences of managers, lean consultants or internal lean champions were excluded due to the role played by these actors in the implementation or the promotion of Lean practices. To ensure consensus on the retained articles, 5% of the identified abstracts were randomly assigned to a second reviewer for assessment using the inclusion criteria. Interrater reliability was subsequently calculated using Cohen’s kappa [27].

**Table 3:** Criteria for included studies

Inclusion criteria	
Publication was written in English or French	
Publication was in a peer-reviewed journal	
Publication was empirical (i.e., involved collection of empirical data either qualitatively or quantitatively or both)	
Publication studied the use of LM practices (i.e., reporting on the use of at least one LM activity)	
Research was undertaken in a healthcare setting (e.g., hospital, ward, department, ancillary services)	

### Data charting and analysis

Full-text analysis was conducted independently by the first author on the retained articles using a data summarising sheet. The sheet was developed and approved by the research team. It recorded essential information including the country of study, language, publication year, publication journal, study setting (e.g., academic hospital, emergency department), reported Lean tools or principles (e.g., value stream mapping, 5S, visual follow-up boards, pull production, Kanban), data collection methods (e.g., interviews, focus groups, observations, surveys), theoretical framework, and staff related findings.

Staff-related outcomes were analysed and synthesised following a three-stage thematic analysis approach [28]. In the first stage, 48 different codes emerged from the findings of the included studies. They were consequently grouped into four different descriptive themes: Morale, motivation and job satisfaction; work intensification, job strain, anxiety, stress and dehumanisation; teamwork, communication and coordination; and learning, innovation and personal development. Analytical themes emerged throughout the data collection and analysis process. They were mapped to the four-fold classification of the findings. Descriptive data from the articles were summarised using numerical counts.

### Risk of bias

The quality of articles was evaluated using Hawker et al.’s Quality Assessment Tool [29]. The tool allows the scoring of papers based on the quality (good (4 points), fair (3 points), poor (2 points) or very poor (1 point) of nine key attributes: abstract and title; introduction and aims; method and data; sampling; data analysis; ethics and bias; findings/results; transferability/generalisability; and, implications and usefulness. Papers can be attributed a maximum score of 36 points (high quality) or a minimum score of 9 points (very low quality). To complement the tool, Lorenc et al. [30] presented a tiered classification of articles depending on their overall quality score: “high quality” (30–36 points), “medium quality” (24–29 points), and “low quality” (9–24 points). This classification was subsequently adapted by J Braithwaite, J Herkes, K Ludlow, L Testa and G Lamprell [31] slightly reducing the cut-off score for low quality articles to 23 instead of 24 points which increased the transparency of the tool. Quality assessment was conducted to indicate the level of confidence with which findings should be taken. Given the emerging nature of this area of study, quality scores were not used to exclude articles from the review.

## Results

**Fig. 1:**
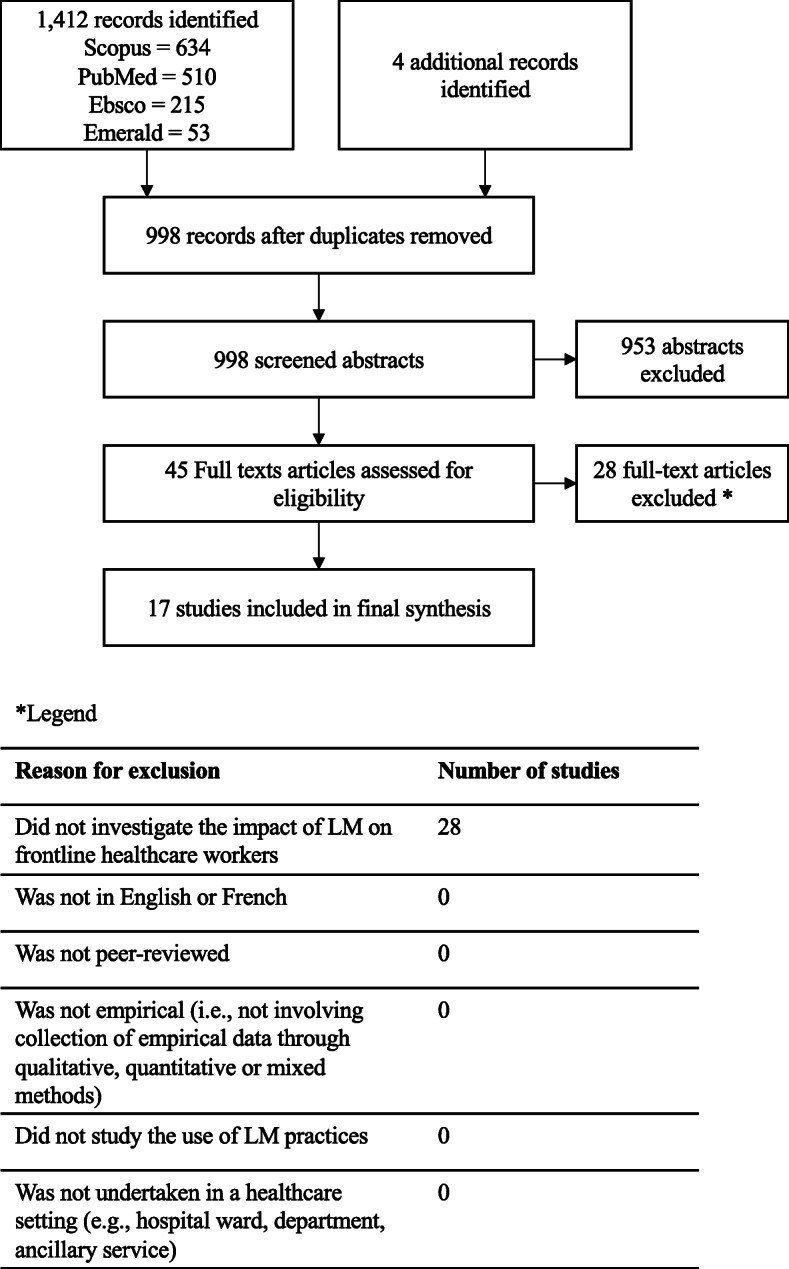
Systematic Review Search Strategy

Out of 998 identified abstracts, 953 were excluded for not meeting the inclusion criteria at title/abstract stage. The Cohen’s Kappa for the 5% randomly assigned abstracts was 0.78, indicating a substantial agreement between reviewers. The full-text review of the articles corresponding to the remaining 45 abstracts resulted in the exclusion of 28 studies for not investigating the impact of LM on frontline healthcare workers (i.e., not discussing effects of LM on staff, not reporting on how staff experienced LM interventions, or discussing staff outcomes as an incidental or secondary finding). In total, 17 studies were included in the final analysis [32–48]. Figure 1 is a graphic representation of the search strategy results using the PRISMA flow chart [49].

The articles included in the review (*n* = 17) were published between 1995 and 2018, with the majority being published in 2014 (*n* = 4, 23.5%) [42, 43, 45, 47] and 2018 (*n* = 4, 23.5%) [32, 35, 38, 48]. All the publications were in English, except one in French [38], and the largest proportion of the studies (*n* = 5, 29.4%) were conducted in Sweden [37, 39, 44–46]. Over half of the publications were in health services journals (*n* = 9, 53%) [32, 33, 35, 37, 39, 42, 45, 48, 50]*.* Five studies (29.4%) were published in nursing [40, 47], surgical [34] and quality in healthcare journals [44, 46]. Only three studies (17.6%) were published in a management or social science journal [38, 41, 43]. The full list of journals is presented in Table 4.

**Table 4:** List of journals included in the review

Journal name	Research field	Number of publications
BMC Health Services Research	Health services research	3
Journal of Nursing Administration	Nursing	2
Quality Management in Healthcare	Quality management	2
American Journal for Health-System Pharmacy	Health services research	1
Global Health Action	Health services research	1
Health Services Management Research	Health services research	1
Journal of Hospital Administration	Health services research	1
Journal of the American College of Surgeons	Surgery	1
The International Journal of Human Resource Management	Management/social science	1
Labour & Industry: a journal of the social and economic relations of work	Management/social science	1
Management et Avenir Santé	Management/social science	1
Journal of Medical Internet Research Human Factors	Health services research	1
Eastern Mediterranean Health Journal	Health services research	1
Total		17

Most of the studies used either qualitative research methods (*n* = 8, 47.1%) [38, 41–44, 46, 48, 50] or quantitative (*n* = 7, 41.2%) [32, 34, 35, 37, 40, 45, 47], with two studies (11.8%) using a mixed-methods approach [33, 39]. Case studies were the most common research design (*n* = 10, 58.8%) [32, 37–39, 41–43, 46, 48, 50], followed by Pre/Post evaluation studies (*n* = 7, 41.2%) [33–35, 40, 44, 45, 47]. Seven studies (41.2%) used a theoretical framework to provide a conceptual foundation for their findings [32, 37–40, 44, 45].

Regarding the research sites, most of the studies were conducted in acute care settings (emergency departments (*n* = 7, 41.2%) [39, 42–45, 47, 48], operating theatres (*n* = 2, 11.8%) [34, 38], and intensive care units (*n* = 1, 5.9%) [34, 38]. Most of the studies (*n* = 13, 76.5%) reported on the use of multiple Lean techniques simultaneously [33–35, 38–41, 43–48, 50]. Only two of the studies did not make any mention of the Lean techniques used in the examined sites [32, 42]. Visual management was the most reported LM technique (*n* = 9) [37–40, 43–46, 48], followed by workspace redesign (*n* = 6) [35, 38, 39, 43, 44, 48], standardisation (*n* = 5) [34, 37–40], and value stream mapping (*n* = 4) [33, 37, 44, 45]. Descriptive information on the studies and a summary of their findings are presented in Additional file 2.

### Risk of bias and quality assessment

Hawker et al.'s (2002) Quality Assessment Tool [29] was used to evaluate the quality and methodological rigour of the reviewed articles because it is suitable for assessing studies with various designs. Table 5 presents an overview of the quality assessment of the articles reviewed. Articles were classified as either high, medium or low quality based on Braithwaite et al.’s (2017) cut-off values. Detailed quality scores are reported in Additional file 3.

**Table 5:** Quality assessment of included studies

Quality classification^a^	Points scored on the Hawker et al. (2002) [29] Quality Assessment Tool	Number of articles classified in each section
High quality	30–36	7
Medium quality	24–29	6
Low quality	9–23	4

^a^Cut-off values determined by Braithwaite et al. [23]

### Overall findings

#### Morale, motivation and job satisfaction

Nine articles (52.9%) found impacts of LM on staff’s morale, motivation, and job satisfaction. In a recent study, LM was associated with improved morale and job satisfaction amongst primary care physicians and medical assistants in a US not-for-profit clinic [35]. Survey data collected in the clinic suggested higher levels of work-satisfaction and personal motivation at work amongst participants after the LM intervention. The intervention included a physical workplace redesign as well as LM-inspired workflow improvements that were associated with increases in employee engagement and participation in decision making.

Similar results were also reported in a U.S. teaching hospital and were considered to be the result of LM’s philosophical foundations which promote giving employees ownership of their work and valuing their perspective [34]. Employee participation, supportive leadership and regular staff meetings were correlated with improvements in job content (i.e., level of influence at work, opportunities for development, the meaning of work, commitment and recognition) in another study conducted in two Swedish cardiac wards [45]. The bottom-up problem-solving approach at the heart of LM and the use of collaborative tools such as value stream mapping were shown to promote employee participation and were considered catalysts for improved wellbeing when they were supported by other resources and used by all professional groups [37].

Nurses in a private medical centre also indicated increased levels of job satisfaction after LM principles were applied in their telemetry unit [40]. Amongst the reported benefits of this intervention was an 85% reduction in the distances walked by staff members during their shifts. The LM-inspired reform also contributed to a decrease in overtime, allowed nurses to routinely take their breaks and created conditions that enabled them to follow their professional values.

A study conducted in two Swedish hospitals and one health municipality showed that work standardisation and the use of 5S were positively correlated with improved job satisfaction among staff [37]. Similar findings were reported in a Senegalese hospital that used 5S, a method of LM, to declutter and to improve the hygiene and the overall cleanliness of the workplace [50]. In a New Zealand study, morale improvements were also experienced by staff working across three emergency departments that adopted the LM tools of 5S, standardisation and value stream mapping [42].

In Australia, job satisfaction improved after the implementation of LM in two public hospitals as it enabled service workers to benefit from new professional status, greater task variety and access to new career paths [41]. Job reconfigurations undertook as part of the same intervention also allowed individual staff members to gain greater peer recognition which further contributed to improved job satisfaction.

Increased satisfaction of intensive care nurses and pharmacy technicians was reported after LM tools were used to reconfigure the continuous renal replacement therapy workflow at a major academic hospital in the US [33]. In this case, the rise in satisfaction scores was attributed to a decrease in nurses’ workload (measured by the number of phone calls to the pharmacy), as well as enhanced production planning by the pharmacy staff.

#### Work intensification, job strain, anxiety, stress and dehumanisation

Seven of the reviewed studies (41.2%) suggested that LM led to work intensification, job strain, anxiety, work-related stress and dehumanisation. In a Swedish study, the adoption of LM led to a significant imbalance between the job resources at the disposal of staff and their job demands, leading to a deterioration of work conditions over time [37].

Similarly, O’Donnell (1995) critically assessed the impact of a LM-inspired reform on the services staff at two Australian hospitals [41]. His research showed that LM led to considerable work stress and intensification due to the elimination of slack and the amalgamation of professional roles. Higher levels of peer-surveillance were also reported as staff increasingly monitored each other’s performance. Furthermore, multiskilling was criticised for being a façade behind which pressure was put on teams to execute labour-intensive tasks. The author noted that in one of the studied hospitals, the adoption of LM was accompanied by forms of managerial coercion, forcing employees to adhere to the new proposed work organisation by, for example, threats of closure and intensification of work conditions for resisting staff.

Evidence of work intensification was also found in a study examining LM in an Australian emergency department [43]. Even though the increase in workloads was attributed to macro-level issues of budgetary pressures being exercised on public healthcare institutions, the authors indicated that LM could lead to work intensification merely by allowing organisations to increase their service capacity while maintaining the same levels of resources.

More recently, wide-scale survey data collected by Hung and colleagues (2018) showed a significant increase in levels of workplace stress, burnout and emotional exhaustion amongst physicians and non-physicians following the implementation of LM at a large ambulatory care facility [35]. Decreased levels of personal accomplishment were mainly reported among the clinical population indicating a negative self-evaluation of the care-related activities they conducted following the LM intervention. Despite the report of positive effects on engagement, teamwork and participation in decision making, the authors’ results indicate that in the studied context, LM did not seem to improve efficiency without negatively impacting hospital staff. Similar findings were reported in three emergency departments in New Zealand where increased levels of work intensification where reported despite improvements in morale [42].

Looking at the use of LM in a French operating theatre (OT), Mahmoud et al. (2018) revealed LM promotes and embodies thoughts that may lead to the instrumental use and dehumanisation of individuals [38]. Using Honneth’s [51] concept of reification, the authors characterised experiences of dehumanisation in three forms of relationships in which operating theatre nurses were engaged (i.e., with other nurses, with the organisation, with patients). In the OT, reification was associated with staff being solely focused on achieving pre-set goals that they objectify their colleagues in the process. It was promoted at the organisational level when individuals felt reduced to a set of skills and were used instrumentally to achieve organisational goals. Reification was also apparent when the human side of care was relegated to the background as patients became increasingly considered as income-generating resources.

In another study, Zebrowski et al. (2018) explored the impact of LM on the clinical work of emergency nurses and physicians in Canada [48]. The authors found that LM was associated with a decline in morale, an increase in physical, emotional and cognitive stress which exposed the nurses to high risks of developing burnout.

#### Teamwork, communication and coordination

Six of the articles (35.3%) included in the review indicated that LM was positively associated with improvements in teamwork, communication, and coordination amongst staff members. In three case, these improvements were attributed to a physical workplace redesign which involved combining workstations of care team members [35, 39, 44]. The new stations allowed staff to spend less time locating each other while acting as a convenient platform for sharing patient information leading to enhanced communication and collaboration.

Coordination between staff was also improved as a result of work standardisation, continuous flow and the use of a team-based approach in a Swedish hospital. These LM tools were shown to have reduced misunderstandings, errors and duplications [39]. Leadership rounds (i.e. Gemba walks) in a UK hospital were linked to better relationships and teamwork between managers and staff [47]. The rounds provided managers with an in-depth understanding of the challenges faced by the teams. Visual Management tools such as whiteboards also facilitated both synchronous and asynchronous communication between staff and managers [46].

Improvements in teamwork were also self-reported by staff after LM was applied to the perioperative otolaryngologic workflow in an American university hospital [34]. Participants in this study reported improvements in the six dimensions of the validated Safety Attitudes Questionnaire (SAQ) [52], including teamwork, when the survey was administered before and after the LM intervention.

#### Learning, innovation and personal development

Three of the reviewed studies (17.6%) examined the impact of LM on the learning and personal development opportunities available to staff as well as their innovation skills. Survey data collected in an academic operating theatre showed that the implementation of LM had no impact on the intraoperative teaching activities [34]. The authors of this study argued that LM could provide additional high-value training opportunities by increasing the capacity of the operating theatres and reducing low-value, time-consuming activities such as unnecessary or redundant administrative work. However, the authors did not provide any data in support of this hypothesis.

In a Senegalese hospital, the adoption of LM was shown to have helped foster a mutual learning environment in which employees engaged in peer-education activities. These participants highlighted the ways that LM helped them enhance their physical work-conditions [50]. Another study revealed that LM had a significant positive effect on the innovation skills of employees [32]. The study was conducted using a self-administered questionnaire completed by 400 employees working in 11 private and two public hospitals.

## Discussion

Overall, the articles reviewed alternately described the relationship between LM and employee outcomes as positive, negative, or mixed (i.e., both positive and negative in the same setting). On the one hand, LM was found to have helped improve teamwork, communication and coordination between staff [34, 35, 39, 44, 46, 47]. It was shown to potentially provide staff with increased learning and personal development opportunities [34, 36] and was linked to improved innovation skills, morale, motivation and job satisfaction [32–35, 37, 40–42, 45, 50].

On the other hand, LM was correlated with higher levels of stress, job strain, anxiety, work intensification and dehumanisation [35, 37, 38, 41, 43, 48]. The inconsistent outcomes of Lean are exemplified by studies that simultaneously found an association between LM interventions and both positive and negative employee outcomes [35, 37, 41]. Table 6 provides an overview of the review results classifying them according to the studies’ overall assessment of LM’s impact on staff.

**Table 6:** A classification of LM impacts on healthcare workers results

	Staff-related outcomes	Articles
Positive outcomes	Teamwork, communication, coordination	[34, 35, 39, 44, 46, 47]
	Learning, innovation and personal development	[32, 34, 50]
	Morale, motivation, and job satisfaction	[33–35, 37, 40–42, 45, 50]
Negative outcomes	Work intensification, job strain, anxiety, stress and dehumanisation	[35, 37, 38, 41–43, 48]

Beyond the limited range of research conducted on the human outcomes of LM in healthcare, the review also reveals the lack of both methodological diversity and rigour that characterises the existing literature. Most of the included studies (*n* = 10, 58.8%) lacked a theoretical conceptualisation of the staff related outcomes of LM and were constrained by reporting descriptive results with relatively limited analytical reach.

Furthermore, despite examining the use of multiple LM tools and techniques, only one of the studies considered Lean holistically, as an organisational, system-wide approach designed to target waste and improve the production of value [38]. The majority of the studies, instead, focused on assessing the outcomes of using specific Lean-related tools or techniques. Accordingly, a large number of the studies adopted an evaluation design that, while useful, substantially limits the generalisability of the results and the conclusions that can be drawn about the impact of LM on staff. Generalisability was also hindered by the adoption of single case study designs, often conducted in one country, as well as by the absence of theoretical framing of the study or results, or both.

Most of the articles in this review exclusively reported positive employee-related outcomes of LM (*n* = 11, 64.8%), perhaps reflecting what has been described as a persistent bias towards the publication of LM related success stories [20, 23]. In contrast, three studies found that LM was only associated with adverse outcomes for employees [38, 43, 48]. It is important to note that none of these studies was able to identify a causal relationship between LM and negative workforce experiences. Instead, the authors highlighted the importance of considering the role played by broader financial and budgetary constraints to which health systems are subject, as well as how LM tools and practices were implemented within the studied organisations. A closer examination of these two areas has the potential for resolving what seems to be the paradox of LM, in that it was originally described as an approach based on worker engagement and input [1] yet in some instances appears to be detrimental to their wellbeing.

More broadly, this review calls for more critical assessments of LM’s impact on healthcare professionals (Table 7). Such assessments would involve identifying the reasons why LM is associated with positive outcomes in some instances, negative ones in others and sometimes mixed outcomes, simultaneously, within the one setting. It is currently impossible to pinpoint the reasons for these inconsistent outcomes, given the absence of information in the reviewed articles on the context surrounding the adoption and implementation of LM. It is hoped that future researchers use robustly designed comparative studies that would allow for such critical analyses to be conducted. Such studies should favour qualitative research methodologies to capture the context surrounding the use of LM as well as aspects pertaining to its implementation and how it is experienced by staff.

**Table 7:** Key recommendations for future research

Recommendations for future empirical work	
More studies need to consider LM holistically and examine the interactions between its technical and human components.	
Future studies should adopt rigorous and methodologically diverse approaches that favor analytical results over descriptive ones.	
Studies discussing unsuccessful lean implementations or deleterious impacts on the workforce are needed. They can help identify pitfalls that could be avoided during the implementation of LM	
International collaborations and comparative study designs could help identify contextual and organisational factors that may lead to positive, negative or mixed outcomes on staff.	
Studies discussing positive staff outcome should seek to highlight factors contributing to those outcomes that could be replicated in different settings.	

The results of this review are reflective of the broader literature on LM and its impact on staff working in other industries. In a recent review, F Magnani, V Carbone and V Moatti [53] also pointed to the restricted number of studies focusing on the impact of LM on employees. They highlighted the inconsistent nature of the research findings on this topic. Further work that holistically examines LM and encompasses its sociotechnical and human dimensions is therefore crucially needed, especially given the demonstrated potential of this approach that can help increase the capacity and improve the efficiency of health systems.

This review has limitations that should be considered. With a primary focus on the impact of LM on frontline healthcare professionals, the review did not report on findings from research examining the experiences of other professionals working in the health systems (e.g. managers, directors, managers, lean consultants or other staff championing Lean initiatives). The findings of the review were also limited to those of published peer-reviewed journal articles written in English or French. Future researchers may choose to attend to other types of academic and non-academic publications in different languages to identify new information on this topic.

## Conclusion

In conclusion, this review constitutes the first attempt to synthesise and critically reflect on the published academic literature examining the impact of LM on frontline healthcare professionals. The review highlighted the contested and inconclusive nature of the research on this topic. While some researchers identified positive impacts of LM, others found more mixed results. Overall, studies that holistically examine cases of Lean implementation in healthcare by attending to its sociotechnical and human dimensions remain scarce. Future researchers should prioritise qualitative and comparative research designs that can help address what seems to be a persistently underexploited area of empirical research.

## Supplementary Information

**Additional file 1.**

**Additional file 2.**

**Additional file 3.**

## Data Availability

All data generated or analysed during this study are included in this published article and its supplementary information files.

## Abbreviations

LM: Lean management
PRISMA: Preferred reporting items for systematic review and meta-analysis
OT: Operating theatre
SAQ: Safety attitude questionnaire
TPS: Toyota production system
US: United States
UK: United Kingdom
