# Supplementary material for: The impact of lean management on frontline healthcare professionals: a scoping review of the literature
Source: BMC Health Serv Res. 2021 Apr 26;21:383. doi: 10.1186/s12913-021-06344-0 (PMC8074224; doi:10.1186/s12913-021-06344-0)
Supplement: Supplementary file 1 — Additional file 1. [file 12913_2021_6344_MOESM1_ESM.docx]

**Additional file 1:** search string example from Sopus and PubMed

| Database | Query |
| --- | --- |
| Scopus | ( TITLE-ABS-KEY ( "Lean approach"  OR  "Lean process"  OR  "Lean method"  OR  "Lean transformation"  OR  "Lean philosophy"  OR  "Lean principles"  OR  "Lean practices"  OR  "Lean process improvement"  OR  "Lean management"  OR  "Lean healthcare"  OR  "Lean health care"  OR  "Lean thinking"  OR  "Lean production"  OR  "Lean Six Sigma"  OR  "Toyota production system" ) )  AND  ( TITLE-ABS-KEY ( healthcare  OR  "health care"  hospital  OR  "operating theatre*"  OR  "bloc* opératoire*"  OR  "operating room*"  OR  "operating ward"  OR  "surgical theatre*"  OR  "surgical ward*"  OR  surg*  OR  care ) )  AND  ( LIMIT-TO ( DOCTYPE ,  "ar" ) )  AND  ( LIMIT-TO ( LANGUAGE ,  "English" )  OR  LIMIT-TO ( LANGUAGE ,  "French" ) )  AND  ( LIMIT-TO ( SRCTYPE ,  "j" ) ) |
| PubMed | (((healthcare[Title/Abstract] OR “health care” hospital[Title/Abstract] OR "operating theatre*"[Title/Abstract] OR "bloc* opératoire*"[Title/Abstract] OR "operating room*"[Title/Abstract] OR "operating ward"[Title/Abstract] OR "surgical theatre*"[Title/Abstract] OR "surgical ward*"[Title/Abstract] OR surg*[Title/Abstract] OR Care[Title/Abstract]))) AND ((“Lean approach”[Title/Abstract] OR “Lean process”[Title/Abstract] OR “Lean method”[Title/Abstract] OR “Lean transformation”[Title/Abstract] OR “Lean philosophy”[Title/Abstract] OR “Lean principles”[Title/Abstract] OR “Lean practices”[Title/Abstract] OR “Lean process improvement”[Title/Abstract] OR “Lean management”[Title/Abstract] OR “Lean healthcare”[Title/Abstract] OR “Lean health care” [Title/Abstract] OR “Lean thinking”[Title/Abstract] OR “Lean production”[Title/Abstract] OR “Lean Six Sigma”[Title/Abstract] OR “Toyota production system”[Title/Abstract])): |
