# Supplementary material for: The impact of lean management on frontline healthcare professionals: a scoping review of the literature
Source: BMC Health Serv Res. 2021 Apr 26;21:383. doi: 10.1186/s12913-021-06344-0 (PMC8074224; doi:10.1186/s12913-021-06344-0)
Supplement: Supplementary file 2 — Additional file 2. [file 12913_2021_6344_MOESM2_ESM.docx]

**Additional file 2:** Studies included in the review

| Author and year | Aim | Country/ Language | Setting | Lean tools/principles | Data collection method(s) | Staff-related findings | THF | Study design |
| --- | --- | --- | --- | --- | --- | --- | --- | --- |
| Aoun, Hasnan et al. (2018) | Study the relationship between lean practices, soft TQM and innovation skills in Lebanese Hospitals | LBN / English | Private and public hospitals | Not reported | Self-administered survey | Lean had a significant positive effect on the innovation skills of employees. | Theory of constraints and the resource-based view theory | Quantitative and cross-sectional case study |
| Benfield, Brummond et al. (2015) | Apply Lean principles to optimise the administration of Continuous Renal Replacement Therapy (CRRT). | US / English | Academic hospital- intensive care unit (ICU) and pharmacy | A3  Value Stream Mapping (VSM) | Mixed methods- quantitative performance data, qualitative survey | ICU nurses and pharmacy technicians reported increased overall satisfaction with the new CRRT process after the implementation of Lean.  ICU Nurses reported a decrease in workload and pharmacy staff reported enhanced production planning. | N | Pre/Post-test evaluation |
| Collar, Shuman et al. (2012) | Examine the impact of Lean thinking on efficiency, profitability, team morale and educational activities. | US / English | Academic hospital – otolaryngology operating room | 5 whys  Root cause analysis  Swim lane diagram  Task standardisation | Quantitative- survey | Overall teamwork and morale improvements.  No impact on intraoperative learning opportunities because of the Lean intervention. | N | Pre/Post-test evaluation |
| Hung, Harrison et al. (2018) | Explore physician and staff experiences after implementing a series of Lean-inspired workflow changes. | US / English | Not-for-profit ambulatory care facility – primary care departments | 5S  Work setting redesign  Process redesign  Job redesign | Quantitative- survey | Higher levels of engagement, participation in decision making and teamwork were reported.  Higher levels of burnout and increased perception of the workplace as stressful were also reported. | N | Pre/Post-test evaluation |
| Kanamori, Sow et al. (2015) | Assess the impact of Lean tools on care-delivery processes and outcomes as well as their applicability in resource-poor settings. | SEN / English | Regional health centre – multidisciplinary outpatient clinic, pharmacy, and inpatient wards | 5S | Qualitative- interviews | Increased willingness to come to work.  Increased motivation.  Peer-to-peer learning. | N | Single Case study |
| Lindskog, Hemphälä et al. (2016) | Identify to what degree Lean tools impact working conditions for employees and managers in healthcare organisations while considering contextual aspects of the implementation process. | SWE / English | Two academic hospitals and one health municipality | Visual follow-up boards  Standardisation  5S  Value Stream Mapping (VSM) | Quantitative- survey | Standardised work promoted engagement in development and job satisfaction and was not associated with exhaustion.  Participation in decision making increased over time.  5S and standardisation positively affected job satisfaction.  VSM is most effective in promoting participation, engagement, and job satisfaction.  A deterioration in working conditions over time in a context of job resources not being balanced with job demands.  Visual follow up boards negatively affected job satisfaction due to insufficient resources. | Job demands-resources model | Single Longitudinal case study |
| Mahmoud and Angelé-Halgand (2018) | Examine the impact of Lean on medical and allied health professionals | FRA / French | Public hospital operating theatre | Visual management  Continuous flow  Just in time  Pull production  Work setting redesign  Standardisation | Qualitative non-participant observation and interviews | LM contributed to the creation of a reifying environment for nurses. Revealed the appearance of logics of domination generating contempt and humiliation amongst nurses.  Signs of reification of patients. | Reification | Single Case study |
| Mazzocato, Holden et al. (2012) | Examine a Lean inspired intervention in a paediatric accident and emergency department. | SWE / English | Academic hospital – paediatric accident and emergency department | Work setting redesign  Visual management  Takt time management  Standardisation  Continual improvement  First-Time quality  Continuous flow | Mixed methods – quantitative performance data, interviews, non- participant Observation, document analysis | Reduced ambiguity and variation around roles and responsibilities.  Reports of work being narrowly regulated and monotonous.  Improvements in teamwork and coordination between professionals attributed to a physical redesign of workspaces. The interprofessional collaboration did not improve.  Staff reported a feeling of being monitored by flow managers (using visual progress monitoring boards).  Reports of fewer misunderstandings and work duplication. | Spear and Bowen (1999) | Single Case study |
| Nelson-Peterson and Leppa (2007) | Discuss the application of the Virginia mason production system and how it has resulted in increased time for nurses to care for their patients. | US / English | Private medical centre – telemetry unit | Rapid process improvement Workshop  Visual management  Just in time  Kanban  Standardisation  One-piece flow cycle time  U-shaped cell | Quantitative- performance metrics | 85% reduction in walking distance (job-related fatigue).  Staff have routine breaks and lunches.  Decrease in overtime.  Increase in staff satisfaction.  More time to care. | Swanson's theory of caring | Pre/Post-test evaluation |
| O'Donnell (1995) | Critically assesses the impact of Lean-inspired workplace reforms upon hotel service worker from a non-English-speaking background in public hospitals. | AUS / English | Two public hospitals – hotel services | Just in time  job redesign Multiskilling  Teamwork | Qualitative- interviews | Increased job satisfaction and morale due to greater task variety, new professional status, access to career paths, greater recognition by peers.  Reports of increased workload, intensification of labour and increased work-related stress.  Reports of increased peer pressure, a conflict between team-members. | N | Single  Case study |
| Rees (2014) | Describe and contrast the implementation of Lean in three hospitals. | NZL / English | Three hospitals – emergency departments | Not reported | Qualitative- interviews | Morale improvements were reported across the sites.  Improved teamwork.  Work intensification was reported and dealt with differently (absorbed or ignored).  Workplace resistance was reported in the form of disengagement and de-prioritisation of Lean work. | N | Single  Case study |
| Stanton, Gough et al. (2014) | Investigate the implementation of a Lean six sigma project and its impact on critical medical and nursing staff. | AUS / English | Tertiary hospital – emergency department | Visual management  DMAIC  Process redesign  Physical workplace redesign. | Qualitative document analysis, semi-structured interviews. | Reports of work intensification attributed to an increased rate of patient turnover (similar amount of work must be done in shorter time) also linked to global context of external pressure on hospitals. | N | Single Case study |
| Ulhassan, Sandahl et al. (2013) | Understand why organisations adopt Lean and how it affects employees' work. | SWE / English | Acute care hospital – cardiology department and emergency department | 5S  Value Stream Mapping (VSM)  Physical workplace redesign  Job redesign  Visual management  Problem-solving  Work process redesign  Education/training | Qualitative – Non-participant observations, document analysis, semi-structured interviews | Improvements in communication and coordination among staff attributed to physical work redesign.  Improved perception of work atmosphere reported by managers and staff.  High ownership and engagement attributed to problem-solving workshops. | Holden conceptualisation of Lean (Holden 2011) | Pre/Post-test evaluation |
| Ulhassan, von Thiele Schwarz et al. (2015) | Examine how Visual Management affect staff in healthcare settings. | SWE / English | Acute care hospital – cardiology wards | Visual management in the form of suggestion whiteboard | Qualitative data - semi | The use of VM helped staff express their ideas freely and anonymously.  VM allowed for smoother interactions and better communication between various teams.  Staff reported a sense of empowerment and greater control over their work.  The use of VM was also considered burdensome overwork by staff and was abandoned. | N | Single Case study |
| Ulhassan, von Thiele Schwarz et al. (2014) | Examine the impact of Lean on the psychosocial work environment. | SWE / English | Acute care hospital – emergency department and two inpatient cardiac wards | 5S  Value Stream Mapping (VSM)  Continuous improvement and visual management  Job/work process redesign  Teamwork  Education/training | Quantitative questionnaire | In wards 1 and 2, improvements were reported in the domains of "work organisation and job content" and "interpersonal relations and leadership" - attributed to active employee participation, supportive leadership, and regular meetings.  In the ED, deterioration was reported in the domains of "work organisation and job content" and "interpersonal relations and leadership" – attributed to the less frequent staff meeting and decreased participation.  In ward 2, deterioration was reported in the domains of "role conflict" and "social support" – attributed to the failure of problem-solving activities and lack of social support amongst employees. | The demand control model | Pre/Post- Test evaluation |
|  |  |  |  |  |  |  |  |  |
| Vose, Reichard et al. (2014) | Examine how Lean was used to improve performance in an overcrowded emergency department. | UK / English | Community hospital – emergency department | A3  Gemba Walking | Quantitative performance data – pull times, | Improved sense of control over workload was reported due to process changes after the intervention.  Reports of improved relationships and teamwork between managers and staff members attributed to Gemba walks. | N | Pre/Post-test evaluation |
| Zibrowski, Shepherd et al. (2018) | Explore how Lean impacts the clinical work of emergency nurses and physicians | CAN / English | 2 emergency department at 1 teaching hospital | Work setting redesign  Visual management | Interviews | Reports of physical, emotional, and cognitive stress.  Sense of declined morale.  Reports of diminished autonomy over work.  Perceived diminished value of nurses' role within the department due to unintentional deskilling.  Reports of work intensification.  High risk of developing burnout syndrome. | N | Single Case study |

Key: THF: Theoretical Framework, US: United States, UK: United Kingdom, SEN: Senegal, SWE: Sweden, AUS: Australia, NZL: New Zealand, FRA: France, LBN: Lebanon.

Source: Author's conceptualisation
