# Supplementary material for: The impact of lean management on frontline healthcare professionals: a scoping review of the literature
Source: BMC Health Serv Res. 2021 Apr 26;21:383. doi: 10.1186/s12913-021-06344-0 (PMC8074224; doi:10.1186/s12913-021-06344-0)
Supplement: Supplementary file 3 — Additional file 3. [file 12913_2021_6344_MOESM3_ESM.docx]

**Additional file 3:** quality assessment of included studies

| **Study** | **Abstract/Title** | **Introduction/aims** | **Methods & Data collection** | **Sampling** | **Analysis** | **Ethics/bias** | **Results** | **Transferability** | **Implications** | **Total** |
| --- | --- | --- | --- | --- | --- | --- | --- | --- | --- | --- |
| Aoun, et al. ^28^ | 4 | 2 | 3 | 2 | 3 | 2 | 3 | 2 | 3  No mention of policy and practice implications | 24 |
| Benfield et al. ^35^ | 3 | 3 | 3 | 1 | 2 | 3 | 3 | 2 | 2 | 22 |
| Collar, et al. ^14^ | 4 | 3 | 4 | 1 | 3 | 3 | 3 | 2 | 4 | 27 |
| Hung, et al. ^32^ | 4 | 4 | 4 | 3 | 3 | 3 | 4 | 3 | 2 | 30 |
| Kanamori, et al. ^33^ | 4 | 4 | 3 | 3 | 3 | 4 | 4 | 3 | 4 | 32 |
| Lindskog, et al. ^23^ | 3 | 4 | 4 | 3 | 4 | 3 | 4 | 3 | 3  No mention of future research | 31 |
| Mahmoud and Angelé-Halgand ^29^ | 3 | 4 | 4 | 3 | 3 | 3 | 4 | 4 | 4 | 32 |
| Mazzocato, et al. ^25^ | 4 | 4 | 4 | 3 | 3 | 2 | 4 | 4 | 3  No mention of policy and practice implications | 31 |
| Nelson-Peterson and Leppa ^24^ | 3 | 2 | 2 | 1 | 2 | 1 | 3 | 2 | 1 | 17 |
| O'Donnell ^34^ | 3 | 3 | 3 | 3 | 2 | 1 | 3 | 3 | 2 | 23 |
| Rees ^30^ | 3 | 3 | 3 | 3 | 3 | 2 | 4 | 3 | 2 | 26 |
| Stanton, et al. ^36^ | 3 | 4 | 3 | 3 | 2 | 2 | 4 | 3 | 4 | 28 |
| Ulhassan, et al. ^26^ | 3 | 3 | 4 | 3 | 4 | 2 | 4 | 3 | 3  No mention of policy and practice implications | 29 |
| Ulhassan, et al. ^40^ | 3 | 3 | 4 | 3 | 3 | 2 | 4 | 3 | 4 | 29 |
| Ulhassan, et al. ^27^ | 4 | 3 | 4 | 3 | 3 | 3 | 4 | 3 | 4 | 31 |
| Vose, et al. ^39^ | 3 | 2 | 2 | 2 | 2 | 1 | 3 | 2 | 2 | 16 |
| Zibrowski, et al. ^38^ | 4 | 4 | 3 | 3 | 4 | 2 | 4 | 3 | 4 | 31 |
